# Do Maternal Stress and Depressive Symptoms in Perinatal Period Predict the Lactation Mastitis Occurrence? A Retrospective Longitudinal Study in Greek Women

**DOI:** 10.3390/diagnostics11091524

**Published:** 2021-08-24

**Authors:** Maria Dagla, Calliope Dagla, Irina Mrvoljak-Theodoropoulou, Dimitra Sotiropoulou, Aikaterini-Taxiarchoula Kavakou, Eleni Kontiza, Evangelia Antoniou

**Affiliations:** 1 Day Center for the Care of the Mental Health of Women (Perinatal Mental Health Disorders), Non-Profit Organization “FAINARETI”, 17121 Athens, Greece; info@fainareti.gr (C.D.); imrvoljak@hotmail.com (I.M.-T.); dswtir@gmail.com (D.S.); kavakoukat@gmail.com (A.-T.K.); helenakontiza@gmail.com (E.K.); lilanton@uniwa.gr (E.A.); 2 Department of Midwifery, University of West Attica, 12243 Athens, Greece

**Keywords:** lactation mastitis, maternal stress, depressive symptoms, EPDS, PASS, perinatal period

## Abstract

Background: The aim of this study is to investigate whether symptoms of anxiety and depression disorders in women during the perinatal period predict the occurrence of lactation mastitis. Methods: This is a retrospective longitudinal study of 622 Greek women who were monitored from pregnancy until the first year postpartum (during the period January 2015–May 2018). The Edinburgh Postnatal Depression Scale (EPDS) and the Perinatal Anxiety Screening Scale (PASS) were administered at four time points: (a) 24th–28th gestation week, (b) 34th–38th gestation week, (c) 6 weeks postpartum, and (d) 12 months postpartum. Multivariate binary logistic regression analyses were performed. Results: Results showed that (a) increased EPDS (*p* < 0.02) and PASS (*p* < 0.05) scores during the last period before birth, (b) increased EPDS score at 6 weeks postpartum (*p* < 0.02), (c) PMS symptoms (*p* < 0.03), (d) traumatic life events during the last year (*p* < 0.03), and (e) the existence of a history of psychotherapy (before pregnancy) (*p* = 0.050) appear to be the psycho-emotional factors that can predict the possible occurrence of lactation mastitis in a breastfeeding mother. Conclusions: The association between women’s poor mental health and the occurrence of a physical health problem, such as lactation mastitis, is recognized. This study highlights the important role of early and timely detection of perinatal mental health disorders.

## 1. Introduction

Breastfeeding is the optimal nourishment for an infant’s physical growth and development. The benefits of breastfeeding to the baby, mother, and society are many and varied. Studies show that breastfed infants have a lower incidence of infections and a reduced risk of acute lymphoblastic leukemia and sudden infant death syndrome [1,2,3]. Additionally, breastfeeding mothers are protected against the risk of breast and ovarian cancer and cardiovascular diseases [1]. Although breastfeeding is a natural process, it is often accompanied by various problems. Lactation mastitis, injured nipples, unsatisfactory milk production, and insufficient weight gain of the newborn are conditions that we know can be obstacles for the initiation of breastfeeding and hamper its smooth course and lead to an unpleasant breastfeeding experience for mothers [4,5]. The percentage of new mothers who complain that they have difficulty breastfeeding ranges from 20% to 80% internationally [6,7,8]. The above pathological conditions can occur at any time during lactation and are due to various causes.

More specifically, lactation mastitis is usually caused by a pathogenic microorganism, with *Staphylococcus aureus* being the most common [9]. The microorganism manages to enter and colonize the breast through the injured nipples. In addition, lactation mastitis can be caused by the overproduction or irregular removal of milk from the breast [10]. Significant risk factors for lactation mastitis that have been recognized are previous mastitis during breastfeeding, breast trauma, latch problems ≤8 weeks post-delivery, milk overproduction, blocked duct, cracked nipple, use of nipple shields, breast pumps, and other factors [11]. The incidence of mastitis among breastfeeding women ranges between 10% (e.g., in the USA) [12] and 24% (e.g., in Australia) [13]. Although a woman can develop mastitis throughout the lactation period, this seems to happen more often in the first 6–8 weeks postpartum [14].

Lactation mastitis is an inflammatory condition of the breast and is characterized by symptoms such as swelling, redness, tenderness, pain, debilitation, and shivers [15]. These symptoms negatively affect the experience of breastfeeding, and, very often, women end up stopping breastfeeding due to these and/or other difficulties [16]. Many women have reported that they thought breastfeeding would be a simpler process, and when faced with such difficulties, they stopped breastfeeding and experienced feelings of guilt and low self-esteem, believing that they had failed in their role as mothers [17]. This shows that women evaluate their role, as mothers, according to whether they breastfeed successfully. Therefore, breastfeeding problems, such as lactation mastitis, can undermine mothers’ perceptions of their ability to cope with their parenting role. When they fail to manage these problems, they feel hopeless and inadequate as mothers and have low self-esteem [17,18]. Such feelings have been associated with an increased risk of postpartum depression or other postpartum disorders [19].

The perinatal period, through the interaction of a number of hormonal, psychological, and social factors, has been linked to the onset of certain mental disorders. Depression and anxiety are the most common mental health problems in pregnancy. About 12% of women are depressed, and 13% have significant stress at some point during pregnancy. These difficulties often co-occur. Depressive and anxiety symptoms also occur in 15–20% of women in the first year after birth and can be mild, moderate, or severe [20,21]. Anxiety disorders during pregnancy and the postpartum period mainly include panic disorder, generalized anxiety disorder, obsessive–compulsive disorder, post-traumatic stress disorder, and tokophobia (intense fear of childbirth). Perinatal mental disorders can have significant effects on the health and well-being of not only the mother but also her partner and the development of the fetus/baby [20,21,22].

The consequences of developing a physical problem during lactation, such as mastitis, have been associated with a reduced duration of breastfeeding [23]. The pain that the mother feels and the difficulties she faces during breastfeeding, which often lead to its early cessation, have been associated with higher scores in psychometric tools that detect a mental health disorder, resulting in the poorer mental health of women in the perinatal period [24,25]. The opposite direction of the relationship between breastfeeding and mental health has also been investigated, with studies showing that excessive stress and depressive symptoms negatively affect the continuation of breastfeeding and contribute to its early cessation [25,26]. It is worth noting that there have been studies investigating the association between depression and inflammation in general, following the biological path [27]. These studies have reported the presence of increased inflammatory cytokines in people with major depressive disorders. Cytokines are a group of peptides used by immune cells to communicate with each other and with their environment. Studies show that they play a key role in controlling the immune response, inflammation, hematopoiesis, healing, and regulating the normal function of cells in the body. Other studies [28,29] have reported that, compared with non-depressed individuals, both patients and healthy individuals with major depression have been found to exhibit all of the key features of inflammation. Correspondingly, it has been shown that both acute and chronic stress are associated with increased availability of pro-inflammatory cytokines and decreased availability of anti-inflammatory cytokines. Thus, it is evident from these results that depression and anxiety could possibly affect the function of the immune system, which, in turn, could affect the risk of infection.

The relationship between lactation mastitis, an inflammatory condition, and depression has been investigated to a limited extent and mainly through studies exploring the relationship between physical health difficulties/problems and depression [18,19,30,31]. The opposite direction of the relationship between women’s mental health in the perinatal period and the occurrence of lactation mastitis has not been investigated as far as we are aware. This relationship came to the attention of the midwives of the Day Center for the Care of the Mental Health of Women (Perinatal Mental Health Disorders) in Greece. During their contact with women participating in the Day Center’s perinatal intervention health program, the midwives noticed that lactation mastitis occurred more often in women who exhibited mental health difficulties. This observation led to the hypothesis of the present study; women that show depressive or anxiety symptoms during the perinatal period will suffer from lactation mastitis more often than women who do not show such symptoms.

Therefore, the aim of this study is to explore whether symptoms of anxiety and depression disorders in women during the perinatal period predict the occurrence of mastitis during lactation, specifically within the first year postpartum.

## 2. Materials and Methods

### 2.1. Study Population

This research is a retrospective longitudinal study. The sample of the study population was obtained from a primary mental health facility in Athens, the Day Center for the Care of the Mental Health of Women (Perinatal Mental Health Disorders), that has been operated by the non-profit organization “FAINARETI” since 2009 and is supervised by the Greek Ministry of Health. The study was conducted on a sample of 622 women who used the Day Center’s services from January 2015 to May 2018. Data were obtained from the Day Center’s archives. The inclusion criteria were the following: (a) the women should have completed the Day Center’s intervention program, (b) there should be sufficient recorded data for each subject (e.g., a complete health history), and (c) the data should be written in Greek.

### 2.2. Data Collection

The innovative perinatal intervention health program of the Day Center, providing services from pregnancy up to the first year postpartum, offers midwifery-led education, counseling, and support, along with timely screening for perinatal mental health disorders to all women and, at the same time, timely treatment and counseling by mental health professionals to those women and their partners when considered necessary.

**Figure 1:**
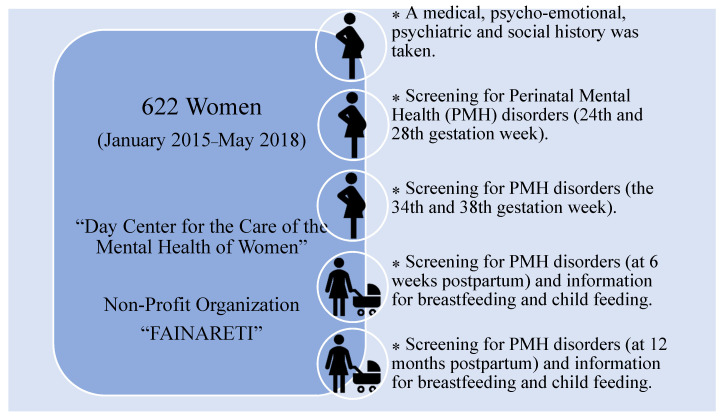
Flowchart regarding the data collection.

Before the start of the intervention program, a comprehensive medical, psycho-emotional, psychiatric, and social history is taken, which is then supplemented with data at 6 weeks and 1 year postpartum (mainly with information on breastfeeding and child feeding). Both the screening for symptoms of a perinatal mental health disorder (mainly anxiety and depression disorder) and the assessment of these disorders are repeated at four time points before and after childbirth: (a) at the beginning of the program (approximately between the 24th and 28th gestation week), (b) during the last period before birth (approximately between the 34th and 38th gestation week), (c) at 6 weeks postpartum, and (d) at 12 months postpartum (Figure 1).

### 2.3. Screening Tools

The screening tools that were used at the four aforementioned time points included: (a) The Edinburgh Postnatal Depression Scale (EPDS) and (b) the Perinatal Anxiety Screening Scale (PASS). The EPDS [32] is a widely used tool that detects pathological symptoms associated with prenatal and postnatal depression within the previous 7 days. The Greek version of the tool was used in the present study [33]. Statistical analysis suggested a cut-off point of 11/12, with a sensitivity of 90% and a specificity of 97.2% [33]. In this study, the average Cronbach’s alpha was 0.86. PASS [34] is an acceptable tool for recognizing anxiety that is used prenatally and postnatally. It has a four-factor structure described as (1) acute anxiety and adjustment, (2) general worry and specific fears, (3) perfectionism, control, and trauma, and (4) social anxiety. PASS has not been culturally adapted to the Greek population. However, for the purposes of the Day Center, it was translated into Greek by two Greek–English bilingual researchers, reviewed by an accredited translator, and back-translated by an English–Greek bilingual researcher. Afterward, it was back-translated to English by another bilingual researcher to confirm its accuracy. As a score of 26 or above is defined as a cut-off score, 68% of women with a diagnosis of an anxiety disorder were identified [34]. In the present study, Cronbach’s alpha reached 0.93.

### 2.4. Ethical Issues

All women who participated in this study were informed of the purposes of the Day Center and the analysis of their information in a research context at the beginning of the Day Center’s program. All women were given an information letter and a consent form as well as time to ask questions for clarification and were all informed that they could withdraw their consent at any time. Oral and written consent was obtained from all women who attended the Day Center program so that the information in their files could be used for research purposes. The women’s names were coded on the history and psychometric tools sheets they had completed, and they were all assigned a protocol number. All data were secured in a safe place accessible only to the Day Center’s healthcare professionals, some of whom constitute the research team of the present article. This research study was approved by the Research Ethics Committee of the Non-Profit Organization “FAINARETI” (Ref. Number 91/17.09.19).

### 2.5. Analysis

Quantitative variables were described as absolute frequencies (*n*) and relative frequencies (%). Logistic regression analyses were performed in order to predict the factors associated with the occurrence of mastitis. Statistical significance was set at 0.05, and data analyses were performed using the Statistical Package for Social Sciences (SPSS) version 22.0. The demographic factors included: maternal age, education, marital status, and monthly income. The perinatal and health history characteristics included: the occurrence of premenstrual syndrome (PMS) symptoms, conception through in vitro fertilization (IVF) procedure, smoking before or during pregnancy, the type of delivery, the woman’s satisfaction with her delivery, the neonate’s weight, and the neonate’s admission to the neonatal intensive care unit (NICU). Mental health variables included: the women’s mental health status, whether the women were experiencing symptoms of mental disorders, whether they were receiving psychotherapy during the perinatal period at the Day Center or had received psychotherapy in the past (before pregnancy), if they were taking psychotropic medications, and if they had experienced a traumatic life event in the last year. All the above variables, as well as the EPDS and PASS scores collected at four time points (from pregnancy to the first year postpartum), were used in a binary logistic regression model in order to identify the factors that could predict the occurrence of mastitis.

Any breastfeeding was defined as feeding with breast milk, with or without any other type of food or drink, including breast milk substitutes (non-human milk and formula), and exclusive breastfeeding was defined as feeding with only breast milk and no other form of foods or liquids, except for oral rehydration solutions, drops, and syrups (vitamins, minerals, medicines), according to the definition of WHO [35]. PMS symptoms were defined as difficulties women can experience in the weeks before their period, such as abdominal pain, headache, fatigue, mood swings and irritability or anger, depressed mood, trouble falling asleep (insomnia), and breast tenderness. No objective or specific definition was given to traumatic life events. As an event can have traumatic mental rather than physical consequences for a person and because all traumatic events are not equally traumatic or do not have the same consequences for all people, in this study, the interpretation of what was considered a traumatic life event was made by the participants themselves. The question they were asked was: “Has anything traumatic happened in your life in the last year?”. The variable “risk factors in mental health history” encompassed factors such as a family history of mental health disorders, the experience of symptoms of mental health disorders in the past, relationship problems with their partner/domestic violence, and poor socio-economic situation/unemployment. In the binary logistic regression model, only statistically significant relationships are reported.

## 3. Results

### 3.1. Study Characteristics

The demographic, perinatal, and breastfeeding characteristics of a total of 622 women who participated in this study are presented in Table 1. The mean age of the women was 32.9 ± 3.9 (SD) years, with the majority being married or living with a partner (*N* = 598, 96.1%). The monthly income was 501–1000 euros for approximately half of the participants (*N* = 288, 46.3%), while a significant percentage of them (*N* = 416, 66.9%) had completed at least undergraduate studies. Most of the women had conceived naturally (*N* = 569, 91.5%). The sample of women was divided into two according to the type of delivery. Half of the women gave birth with vaginal delivery (*N* = 312, 50.2%) and the other half with cesarean section (*N* = 310, 49.8%). Most of the women reported that they were satisfied with their delivery (*N* = 452, 74.5%). One-third of the women smoked before pregnancy (*N* = 222, 35.7%), and a very small percentage reported that they continued to smoke during pregnancy (*N* = 49, 7.9%). In their majority, the neonates were born with a birth weight of 2500–3500 g (*N* = 421, 67.8%), and, from the total number of neonates born, a small percentage was admitted to the NICU (*N* = 93, 15.0%) (Table 1). The vast majority of women (*N* = 602, 96.8%) started breastfeeding in the hospital, either exclusively (*N* = 444, 71.4%) or using mixed feeding (*N* = 158, 25.4%). A similar percentage of women continued to breastfeed (*N* = 606, 97.4%) until the end of the 1st month postpartum, with a very small increase being recorded in the percentage of exclusively breastfeeding mothers (*N* = 472, 75.9%). Regarding breastfeeding duration, a very large percentage of women continued breastfeeding beyond 7 months until the end of the 24th month postpartum (*N* = 452, 72.4%). Additionally, more than half of the women who participated in the present study continued to breastfeed without having given any artificial milk (only complementary foods) (*N* = 361, 58.1%) from the 7th to the 24th month postpartum. The percentage of women in our sample who reported having at least one case of mastitis was 16.2% (*Ν* = 101) (Table 1).

### 3.2. Mental Health Characteristics

Table 2 presents the mental health characteristics of our sample. As can be observed, more than half of the women in the sample (*N* = 395, 63.5%) either had a risk factor in their mental health history (*N* = 295, 47.4%) or had increased scores on the psychometric tools, approximately between the 24th and 28th gestation week. About half of the women in the sample (*N* = 283, 45.5%) did not show any symptoms of mental health disorders during the perinatal period, while the other half showed symptoms of anxiety, depression, or both (*N* = 339, 54.5%). Half of the participants (*N* = 276, 44.4%) reported that they had received psychotherapy at some point in their lives (before pregnancy), and the percentage of participants who received psychotherapy during the perinatal period was quite small (*N* = 72, 11.6%). Additionally, 131 (20.9%) participants reported that they had experienced a traumatic life event in the last year (Table 2).

### 3.3. Multivariate Binary Logistic Regression Analyses

In this study, binary logistic regression analyses were used to analyze the data. Multiple analyses showed that mastitis seemed to be predicted by (a) increased EPDS and PASS scores obtained during the last period before birth (between 34th and 38th gestation week), (b) increased EPDS score at 6 weeks postpartum, (c) PMS symptoms, (d) traumatic life events during the last year, and (e) a history of psychotherapy or contact with a mental health specialist in the past (before pregnancy). All these independent variables showed a statistically significant association with lactation mastitis (Table 3).

For each unit of increase in EPDS score obtained during the last period before birth (between the 34th and 38th gestation week), the risk of developing lactation mastitis appeared to increase by 12% (*p* < 0.02), with 4.4% of the variance explained. During the same period, for each unit of increase in PASS score, the probability of lactation mastitis increases by 6% (*p* < 0.05). This independent variable explained almost the same proportion of the variance as the EPDS score (4.3%). An increased EPDS score at 6 weeks postpartum seemed to be associated with an increased risk for lactation mastitis of 8% (*p* < 0.02) for each unit of EPDS score. The proportion of the variance explained was the highest among all independent variables (10.3%). For each additional symptom of PMS, the probability of occurrence of lactation mastitis seemed to increase by 14% (*p* < 0.03), with 1.4% of the variance explained. Those who experienced a traumatic life event during the last year seemed to have a more than 50% higher risk (60.1%, *p* < 0.03) of developing lactation mastitis. The proportion of the variance explained was 2.4%. Lastly, those who had to contact a mental health specialist in the past were almost twice as likely to develop lactation mastitis (*p* = 0.050) than those who did not seem to need a mental health specialist’s support, with 1.4% of the variance explained (Table 3).

**Figure 2:**
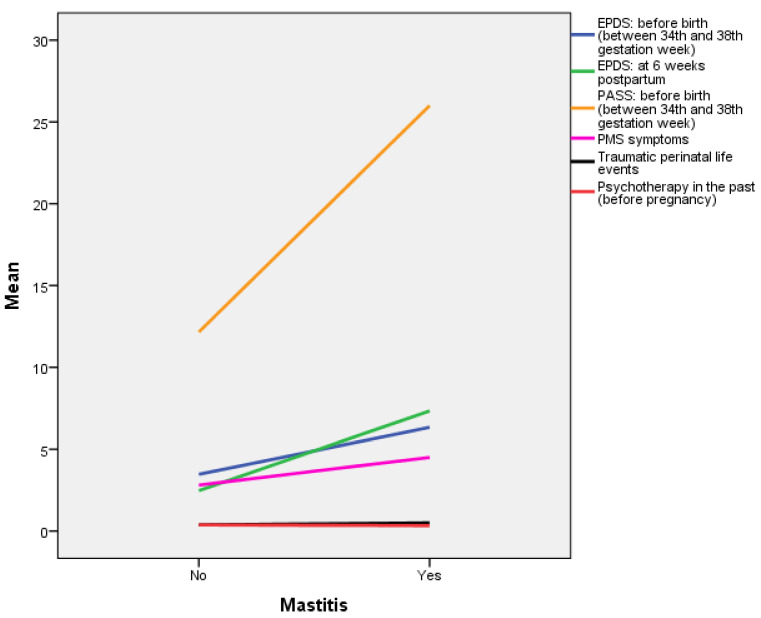
Mean values of the independent variables that seem to predict lactation mastitis. * The variables “traumatic life events” and “psychotherapy in the past” cannot be adequately demonstrated in Figure 1 because they are dichotomous.

In order to graphically present the statistically significant variables that seem to predict lactation mastitis as they emerged from the binary logistic regression analyses, we proceeded to analyze their mean values (Figure 2). According to the mean values, women who reported having lactation mastitis had a higher score on EPDS [*M*(yes) = 5.81, *M*(no) = 4.00] and on PASS [*M*(yes) = 18.15, *M*(no) = 13.28] during the last period before birth (approximately the 34th–38th gestation week) than those who did not report having lactation mastitis. They also had an increased EPDS score at 6 weeks postpartum [*M*(yes) = 6.48, *M*(no) = 4.33], had PMS symptoms [*M*(yes) = 4.06, *M*(no) = 3.61], reported some traumatic life events in the last year [*M*(yes) = 0.22, *M*(no) = 0.10], and had received psychotherapy in the past [*M*(yes) = 0.50, *M*(no) = 0.43] (Figure 2).

## 4. Discussion

This study investigates, for the first time and for a longer follow-up period (from pregnancy up to the first year postpartum), whether specific psycho-emotional characteristics in women during the perinatal period predict the occurrence of a physical health problem, such as lactation mastitis. Generally, the relationship between mental health, especially depressed mood, and the occurrence of a physical illness/health problem has not been clearly recognized in medicine. As mentioned in previous studies, insufficient assessment of the correlation between mental illness and other health conditions has possibly led to the underestimation of the impact caused by mental disorders [36].

### 4.1. The Psycho-Emotional Factors which Predict the Occurrence of Lactation Mastitis

Although several authors have linked a woman’s physical health problems during the puerperium to her mood [30,37], this relationship has not been adequately explored in international literature. Some physical health problems, such as mastitis, have been associated with poor mood postnatally, especially in cases where the woman is suffering from a chronic mental health illness and in cases where more than one physical health problem/illness coexist [18], but this relationship has not been particularly explored during the perinatal period. According to the results of this study, the variables such as (a) the woman’s mental health factors, which are defined by (i) increased EPDS (*p* < 0.02) and PASS scores (*p* < 0.05) during the last period before birth (between the 34th and 38th gestation week), (ii) an increased EPDS score at 6 weeks postpartum (*p* < 0.02), and (iii) the existence of a history of psychotherapy (before pregnancy) (*p* = 0.050); (b) PMS symptoms (*p* < 0.03); and (c) traumatic life events (*p* < 0.03) during the previous year of a woman’s life seem to be the psycho-emotional factors that can predict the likelihood of the occurrence of lactation mastitis by the first year postpartum.

### 4.2. The Relationship between Perinatal Mental Health Disorders and Lactation Mastitis

As far as we know, the occurrence of physical difficulty or health problems during breastfeeding has been associated with a greater risk of developing symptoms of mental health disorders in the postpartum period. In particular, some prospective studies have shown that there is a link between the existence of physical health problems in the first 3 months (or later) after childbirth and the onset of depressive symptoms in women at 6 to 12 months postpartum [19,30,37]. In addition, the recent study of Cooklin et al. [18], which aimed to prospectively investigate the contribution of maternal physical health and/or breastfeeding problems to maternal mood, concluded that women exhibiting multiple physical symptoms and breastfeeding difficulties during the first 8 weeks following birth suffered from a significantly poorer mood at 8 weeks postpartum. Despite the undeniable importance and value of these studies, it should be noted that they did not investigate pregnant women and new mothers’ mental health disorders as a potential risk factor influencing the onset of a physical health problem such as lactation mastitis, as this study does, but they researched women’s mental health as the result of a physical health problem. Additionally, these studies mainly investigated women’s depressed mood in the 6th month postpartum; in contrast, this study investigates the role of anxiety and depression symptoms from an early period, i.e., the end of pregnancy, and continues for a long period postpartum (until the end of the first year).

Lactation mastitis has been characterized as an inflammatory condition of the breast, which may or may not be accompanied by infection [13,15]. The exact biological and psycho-emotional processes that take place so that women with increased symptoms of anxiety and depression may experience a case of lactation mastitis are not investigated in this study. However, we can present some reasoning that supports our findings. Women with increased anxiety and depressive symptoms are generally more concerned about breastfeeding and its course [38] and, therefore, report more difficulties with breastfeeding than other women [39], with one of these difficulties being the occurrence of lactation mastitis. Anxiety is known to interfere with the release of oxytocin and prolactin, hormones that promote the milk ejection reflex [40,41]. Therefore, in some cases, the flow of breast milk into the breast and its removal are hindered, a factor that can lead to lactation mastitis, since it is known that the unsatisfactory emptying of the breast in combination with injured nipples are very important risk factors for developing mastitis [42].

We could also argue that these women may not have complied with the necessary hygiene measures in relation to their breasts or hands and, thus, pathogenic microorganisms can be easily transferred to the nipple and cause infection. Such reasoning may be true, especially if we rely on the finding that people with depressive symptoms often adopt poor health behaviors [36]. Another reasoning that could justify the association between women’s mental health disorders and the occurrence of lactation mastitis is based on the fact that mothers with depressive behavior have poor interaction with their baby [43] and are less sensitive to its needs [44]. As a result, the mother sometimes does not realize the baby’s need to breastfeed, finds it difficult to place the baby on the breast, skips meals, or does not complete them. She may also not empty her breast completely and cause the breast milk to stop, which, as mentioned above, is associated with the development of mastitis [42]. Hence, it is possible that these women follow practices that are risk factors for lactation mastitis without realizing it.

The above reasoning associates the poor mental health of women with the occurrence of lactation mastitis based mainly on biological theories and behavioral reactions that apply to women with pathological mental health symptoms, which that may contribute to the development of a pathogenic microorganism and the appearance of a physical health problem such as mastitis lactation. It is known, however, that excessive stress (outside the normal range) and depressive behavior are generally responsible for hormone imbalances [40,45], the improper functioning of the human immune system [46], and the development or deterioration of physical health problems (e.g., cardiovascular problems) [47]. Human mental and physical health are closely linked so that one affects the other [48]. Additionally, international literature has linked breastfeeding to women’s mental health disorders [19,30,31].

Early cessation and failure of breastfeeding, as well as breastfeeding difficulties, have been associated with excessive stress, bad mood, and depressive symptomatology of the new mother [24,25]. More specifically, the occurrence of depression during the third trimester of pregnancy and the first months postpartum has been linked to the early cessation of breastfeeding [49,50]. Conversely, early adverse breastfeeding experiences appear to be a risk factor for developing postpartum depression, especially in mothers with an aggravated mental health history [51]. Additionally, increased postpartum anxiety can affect breastfeeding due to stress responses and hormonal changes that occur in a woman’s body [40]. Although the exact link between breastfeeding and women’s mental health disorders is not yet known, it is believed that there is a two-way relationship between the cessation of breastfeeding and women’s poorer mental health in the perinatal period [24]. This fact shows the importance of the early recognition of breastfeeding problems in order to avoid the disturbance of women’s mental health. It also shows the importance of early detection of the mother’s pathological mental health symptoms in order to avoid the disturbance of the exclusivity and duration of breastfeeding.

### 4.3. The Benefits of Early Detection for Perinatal Mental Health Disorders

The results of this study highlight and support the need for early screening for symptoms of anxiety and depression disorders in order to timely detect women who are likely to develop psychopathology during the perinatal and lactation period. Early screening (starting at pregnancy) can benefit public health in three ways simultaneously, as described below. First, it can contribute to the early detection of women who are at increased risk of developing perinatal mental health disorders. This will allow for timely treatment by a mental health specialist (e.g., through psychotherapy or other methods), thus avoiding, or at least mitigating, the negative consequences of perinatal mental health disorders for the mother, child, and family [43,52] in the short and long run.

Second, it can contribute to the early identification (from pregnancy) of women who are at risk of developing lactation mastitis during breastfeeding so that they receive timely prenatal information, education, and support regarding the values and proper standards of breastfeeding and they are monitored regularly (follow-up) and supported throughout the breastfeeding and lactation period by midwives and other specialized professionals. In this way, the morbidity rates of lactation mastitis will be reduced [53], as will the breastfeeding problems/difficulties reported by mothers, which decrease their pleasure from breastfeeding [54] and negatively affect their mental health and well-being [18,51]. Third, it can help women experience breastfeeding as being less challenging so that they continue to breastfeed, especially exclusively, and breastfeeding becomes a pleasant experience for them. The positive attitude and continuation of breastfeeding will bring the recognized physical and emotional/mental benefits for both the mother and her child [25,55] and will contribute to the development of healthy people.

Along with the early detection of pathological maternal mental health symptoms and their timely treatment, it is worth noting that international research and literature should focus, in a greater degree and more systematically, on the identification and investigation of risk factors responsible for the occurrence of a perinatal mental disorder. There should also be a focus on the examination of the role of early, timely, and preventive innovative interventions that will contribute to the reduction of maternal morbidity in the perinatal period while supporting the families of women suffering from a disorder. The above efforts are of utmost importance if we consider the number of women who suffer or lose their lives due to the occurrence of a mental disorder, either during pregnancy or the first months after childbirth. Moreover, bearing in mind the burden that the consequences of these disorders impose on the health and future of the newborn/infant/child as they are associated with attachment insecurity, impaired cognitive, social, and emotional development, and long-term behavioral problems [56,57,58,59,60] as well as on the balance of the whole family, it is considered necessary to finance early interventions that will target issues such as parenting quality, the mother–infant relationship, social support of vulnerable groups, and the child’s socioemotional development. The financing of policies that will support the need for early identification in all pregnant women and new mothers and not just high-risk mothers, as well as the need for more early interventions and prevention research, especially in socioeconomically disadvantaged populations and low-income countries, is imperative for any country that respects its citizens and aims to safeguard their health.

### 4.4. Limitations

The sample of this study was obtained from a mental health facility, a Day Center, and not a midwifery/obstetric or other health facility/institution. This could potentially be a limitation, especially if a large number of women with mental health problems participated in our sample. Although one would expect that in a mental health facility, the vast majority of women would have experienced pathological mental health symptoms, this was not the case. This Day Center is a community facility for the prevention and early detection of perinatal mental health disorders while providing antenatal education and preparation for childbirth and parenting. For that reason, it caters to all pregnant women, new mothers, and their partners and not only to those who exhibit psychopathology. This is why, as mentioned in the results section, approximately half the women of our sample (*Ν* = 283, 45.5%) had not shown any pathological mental health symptoms during the perinatal period. Therefore, the sample of this study was not limited to a population of women with mental health problems or aggravated mental health histories. Another limitation of this study is that it is a retrospective study, which used existing data originally collected for reasons other than research. Our data were obtained from the Day Center’s records, as mentioned in the “Data Collection” section, and were acquired for the early detection, assessment, and follow-up of mental health disorders in the perinatal period. This study design may have limited our ability to obtain accurate data for other potential confounding factors regarding the relationship between perinatal mental health disorders and the occurrence of lactation mastitis. The next step is to conduct a multi-center prospective cohort study using a larger sample in order to confirm the result of our study. In addition, we cannot claim that this is a representative sample of the Greek population since the participants live in the region of Attica, and there is no sample from the provinces. However, if we take into account that about half of the Greek population live in this region, we can say that the research has value as it was not carried out on a small number of people with the characteristics of a minority but on a large number of people in the Greek population.

## 5. Conclusions

This study demonstrates the psycho-emotional factors that can predict the possible occurrence of lactation mastitis in a breastfeeding mother. At the same time, it highlights the important role of early and timely detection of pathological symptoms of perinatal mental health disorders from pregnancy and for a long time postpartum. Although further research is needed to accurately investigate and elucidate the complex relationship between women’s poor mental health and the occurrence of lactation mastitis, this study encourages the development of specific prevention strategies aimed at improving clinical practice and, thus, the health of women and their children.

## Figures and Tables

**Table 1:** Demographic, perinatal/health, and breastfeeding characteristics.

Demographic Characteristics	*N/M*	*%/SD*	*Range*	
Age	32.9	3.9	21–41	
Education				
High School	110	17.7		
Bachelor’s Degree	416	66.9		
Master’s/PhD	96	15.4		
Total	622	100.0		
Marital Status				
Married	524	84.2		
Not Married	19	3.1		
Single Parent Family	5	0.8		
Living With a Partner	74	11.9		
Total	622	100.0		
Monthly Income				
0–500 euros	158	25.4		
501–1000 euros	288	46.3		
>1000 euros	176	28.3		
Total	622	100.0		
**Perinatal/Health Characteristics**				
Premenstrual Syndrome (PMS) Symptoms	3.68	1.86	0–8	
Pregnancy after In Vitro Fertilization (IVF) procedure				
No	569	91.5		
Yes	53	8.5		
Total	622	100.0		
Smoking Before Pregnancy				
No	400	64.3		
Yes	222	35.7		
Total	622	100.0		
Smoking During Pregnancy				
No	573	92.1		
Yes	49	7.9		
Total	622	100.0		
Type of Delivery				
Vaginal	312	50.2		
Caesarian Section	310	49.8		
Total	622	100.0		
Woman’s Birth Satisfaction				
Not at all/Little	66	9.6		
Neither Satisfied nor Dissatisfied	104	15.9		
Mostly Satisfied	232	35.2		
Completely Satisfied	220	39.3		
Total	622	100.0		
Newborn Weight				
1500–2500	48	7.8		
2500–3500	421	67.8		
>3500	151	24.4		
Total	622	100.0		
Twins	14	2.3		
Newborn’s Admission to Neonatal Intensive Care Unit (NICU)				
No	529	85.0		
<8 days	69	11.1		
8–15 days	13	2.1		
>15 days	11	1.8		
Total	622	100.0		
**Breastfeeding Characteristics**				
Newborn Feeding in the Hospital				
Exclusive Breastfeeding	444	71.4		
Breastfeeding and Formula	158	25.4		
Formula	20	3.2		
Total	622	100.0		
Newborn Feeding at 1st Month				
Exclusive Breastfeeding	472	75.9		
Breastfeeding and Formula	134	21.5		
Formula	16	2.6		
Total	622	100.0		
Any Breastfeeding Duration			0–30	
No Breastfeeding	14	2.3		
≤6 months	156	25.2		
7–24 months	452	72.4		
Total	622	100.0		
Exclusive Breastfeeding Duration or Breastfeeding Duration Without any Formula			0–30	
No Breastfeeding	58	9.3		
≤6 months	203	32.6		
7–24 months	361	58.1		
Total	622	100.0		
Presence of Mastitis				
No	521	83.8		
Yes	101	16.2		
Total	622	100.0		

Note. *M*—mean; *SD*—standard deviation; *N*—frequencies; %—relative frequencies.

**Table 2:** Mental health characteristics.

	*N/M*	*%/SD*
Women’s Mental Health Status		
No history of mental health disorders/No risk factors/ Normal Scores on Psychometric Tools	227	36.5
With Risk Factors in Mental Health History	295	47.4
Increased Scores using Psychometric Tools	100	16.1
Total	622	100.0
Symptoms of Mental Health Disorders		
Without	283	45.5
Anxiety Symptoms	169	27.2
Depression Symptoms	64	10.3
Anxiety and Depression	106	17.0
Total	622	100.0
Psychotherapy During Perinatal Period		
No	549	88.4
Yes	72	11.6
Total	622	100.0
Received Psychotropic Medication During Perinatal Period		
No	617	99.2
Yes	5	0.8
Total	622	100.0
Psychotherapy in the Past (Before Pregnancy)		
No	346	55.6
Yes	276	44.4
Total	622	100.0
Presence of Traumatic Life Events in the last year		
No	492	79.1
Yes	131	20.9
Total	622	100.0

Note. *M*—mean; *SD*—standard deviation; *N*—frequencies; %—relative frequencies.

**Table 3:** Multivariate binary logistic regression analyses for the identification of independent variables that predict lactation mastitis.

	*B*	*S.E.*	*p*	*Exp(B)*	*R²*
EPDS—before Birth (between 34th and 38th gestation week)	0.114	0.046	0.014	1.120	0.044
PASS—before Birth (between 34th and 38th gestation week)	0.058	0.029	0.041	1.060	0.103
EPDS—at 6 Weeks Postpartum	0.084	0.029	0.004	1.088	0.043
PMS Symptoms	0.132	0.059	0.025	1.141	0.014
Traumatic Life Events	0.940	0.418	0.025	0.391	0.024
Psychotherapy in the Past (before pregnancy)	0.636	0.324	0.050	1.888	0.014

*Note*. *B* = logistic coefficient, *S.E.* = standard error of estimate, *p* = significance, *Exp(B)* = exponentiated coefficient, *R²* = assessment of interpretive power.

## Data Availability

Data are contained within the article.
